# Risk Assessment of Silicosis and Lung Cancer Mortality associated with Occupational Exposure to Crystalline Silica in Iran

**DOI:** 10.34172/jrhs.2022.85

**Published:** 2022-06-30

**Authors:** Nafiseh Nasirzadeh, Zahra Soltanpour, Yousef Mohammadian, Farough Mohammadian

**Affiliations:** ^1^Department of Occupational Health Engineering, School of Public Health, Tehran University of Medical Sciences, Tehran, Iran; ^2^Department of Occupational Health Engineering, Faculty of Health, Tabriz University of Medical Science, Tabriz, Iran; ^3^Department of Occupational Health Engineering, Environmental Health Research Center, Research Institute for Health Development, Kurdistan University of Medical Sciences, Sanandaj, Iran

**Keywords:** Crystalline silica, Exposure, Lung cancer risk Systematic review

## Abstract

**Background:** Exposure to crystalline silica has long been identified to be associated with lung diseases. Therefore, the present study aimed to assess the risk of silicosis and lung cancer associated with occupational exposure to crystalline silica in Iran.

**Study Design:** It is a systematic review study.

**Methods:** Different databases were searched, and the Cochrane method was used for the systematic review. Thereafter, cumulative exposure to crystalline silica (mg/m^3^ -y) was calculated in every industry. The relative risk of death from silicosis was performed using Mannetje’s method. Based on the geometric mean of exposure, the lung cancer risk of exposure to crystalline silica was also calculated.

**Results:** As evidenced by the results, worker’s exposure to silica ranged from a geometric mean of 0.0212- 0.2689 mg/m^3^ (Recommended standard by the American Conference of Governmental Industrial Hygienists (ACGIH) was 0.025 mg/m^3^ ), which is generally higher than the occupational exposure limit recommended by National Institute for Occupational Safety and Health (NIOSH), ACGIH, and occupational exposure limits. The relative risk of silicosis was in the range of 1 to 14 per 1000 people, and the risk of lung cancer in workers ranged from 13-137 per 1000 people.

**Conclusion:** Since workers are at considerable risk of cancer due to exposure to silica in Iran, exposure control programs need to be implemented in workplaces to decrease the concentration of silica.

## Background

 Crystalline silica is the second most common mineral, existing in more than 90% of the earth’s crust^1^; therefore, sand, rock, and soil have the most abundant crystalline silica.^2^ Workers are exposed to crystalline silica in mining, smelting, sandblasting, building, and glass industries.^3^ Previously published studies demonstrated that masons, plasters, and miners had the highest exposure to crystalline silica.^4,5^ The respiratory system is known as the primary pathway of exposure to crystalline silica dust.^6^ Exposure to crystalline silica has long been identified to be associated with lung diseases,^7^ such as silicosis which is a well-known lung disease.^8^

 Exposure to higher concentrations of crystalline silica can cause “acute silicosis” which has a high mortality.^9^ “Chronic silicosis” is the most common form of pulmonary fibrosis among crystalline silica-exposed workers.^2^ The studies have suggested that pulmonary fibrosis can elevate the risk of lung cancer.^10^ International Agency for Research on Cancer (IARC) has introduced crystalline silica in the form of quartz or cristobalite as a human carcinogen.^11^ Workers are exposed to a low concentration of crystalline silica in workplaces; nonetheless, they are faced with a significant risk of cancer.^12^

 There are an estimated 23 million crystalline silica-exposed workers in China^13^; moreover, over three million in India^14^ and over two million employees in the United States^15^ are exposed to crystalline silica. Annually, almost 800 workers die from lung cancer as a result of inhaling crystalline silica in Britain.^16^ The published studies have reported that workers in developing countries are exposed to crystalline silica in workplaces.^17^

 Recently, risk assessment has become one of the most important aspects in the management of occupational diseases.^18^ There is a dearth of research on the assessment of the risk of lung cancer due to exposure to crystalline silica.^18-21^ Some studies have collected quantitative exposure data for the estimation of risk, for instance, Mannetje et al in IARC used a quantitative method for estimating the rate of silicosis mortality in six cohort studies and reported that the rate of silicosis mortality was above the risk of 1 per 1000 typically deemed acceptable by the Occupational Safety and Health Administration (OSHA).^22^ In the other study, Steenland et al examined lung cancer in 10 silica-exposed cohorts and indicated that the estimated excess lifetime risk of lung cancer for a worker exposed from age 20 to 65 at 0.1 mg/m^3^ crystalline silica (the permissible level in many countries) was 1.1%-1.7% (The background lifetime risk of death from lung cancer is 3%-6%.).^23^ In Iran, Azari et al assessed the relative risk of death from silicosis and lung cancer in traditional brick production and reported that this risk was in the range of 1-63.6 per 1000 people, and the risk of lung cancer was 124.08 per 1000 people.^24^

 There is not any organized and comprehensive study on the status of exposure to crystalline silica and its health risk in Iranian workplaces. Therefore, the present study aimed to provide a systematic review of exposure to crystalline silica in all silica-related industries, as well as the estimation of the risk of silicosis and lung cancer due to exposure to crystalline silica.

## Methods

###  Search strategy

 All of the available studies in the field of occupational exposure to crystalline silica, including case-control and cohort studies, were provided.The literature search strategy was conducted using the following keywords: «Silica», “Crystalline silica», «Exposure”, «Occupational exposure», “Industrial», «Workplace», «Factory”, and “Iran”. All of the articles that reported the concentration of crystalline silica in air samples, as well as those published in English and Persian languages, were selected for the study. Due to the numerous applications of silica in recent decades, the query was carried out from 2000-2021. The Cochrane review method was used as a guideline for the systematic review.^25,26^ According to this method, PECO (Participants, Exposure, Comparators, and Outcomes) statement is as follows:

- Participants: Humans, who had occupational exposure to crystalline silica
- Exposure: Exposure to crystalline silica in silica-related industries
- Comparators: People exposed to crystalline silica and other people
- Outcomes: Increasing the concentration of crystalline silica in environmental or individual samples

Web of Science (WOS), Scopus, PubMed, Google Scholar, and SID (Scientific Information Database) were selected to implement the search strategy. In addition, the manual inspection of reference lists was used in order to gain access to more articles and reduce bias.

###  Screening of articles

 The screening of articles was performed by title, abstract, and full text of the articles, separately. The inclusion criteria were all articles performed on occupational exposure to crystalline silica in Iran. On the other hand, the exclusion criterion entailed the articles on the biomonitoring of individuals. Moreover, abstracts (without their full-text available online), review and mini-review articles, conference papers, meta-analyses, modeling studies, books, and unpublished studies were excluded.

 We used EndNote X9® (Thomson Reuters, Toronto, Canada) software^27^ to prepare the list of the articles and finally downloaded the full text of the screened articles. In order to reduce the error, search strategies were used by two researchers in this study separately. When there were disagreements, a third researcher was involved.

###  Data extraction

 As illustrated in Table 1, data extraction was performed based on year, monitoring station number, mean and standard deviation concentration of crystalline silica, method of detection, city, occupation, and industrial activity.

**Table 1:** Basic characteristics of the included studies

**Year**	**Monitoring** **station number**	**Mean±SD**	**Method of detection**	**City**	**Occupation**	**Industrial activity**	**Ref**
2000	-	2.62 ± 0.00	XRD	Semnan	Ferrosilicon	Furnace	^28^
2003	-	0.49 ± 0.105	NIOSH7500	Arak	Lead metal mining	The mines	^29^
2004	22	0.057 ± 0.016	NIOSH7500	Kashmar	Mining	The mines	^30^
2007	40	0.86 ± 1.04	NIOSH7500	Hamadan	Crushing	Stone deformation operations	^31^
2007	75	0.008 ± 0.004	Spectrophotometry	Golestan	Wheat flour producing	Food industry	^32^
2008	24	0.01 ± 0.005	NIOSH7500	Khaf	Iron stone- Hammering	Stone deformation operations	^33^
2008	24	1.48 ± 0.39	NIOSH7500	Khaf	Iron stone- Excavation	Mines	^33^
2009	10	0.275 ± NM	NIOSH7602	East-Tehran	Stone cutting and milling	Stone deformation operations	^3^
2009	10	0.343 ± NM	NIOSH7602	East-Tehran	Foundry work	Foundry	^3^
2009	10	0.132 ± NM	NIOSH7602	East-Tehran	Glass manufacturing	Glass manufacturing	^3^
2009	10	0.267 ± NM	NIOSH7602	East-Tehran	Asphalting	Asphalt manufacturing	^3^
2009	10	0.193 ± NM	NIOSH7602	East-Tehran	Construction	Construction	^3^
2009	10	0.261 ± NM	NIOSH7602	East-Tehran	Sand and gravel mining	Sand and gravel production	^3^
2009	10	0.272 ± NM	NIOSH7602	East-Tehran	Sandblasting	Sandblast	^3^
2009	10	0.328 ± NM	NIOSH7602	East-Tehran	Ceramic manufacturing	Tile and ceramic industry	^3^
2009	10	0.160 ± NM	NIOSH7602	East-Tehran	Bricks manufacturing	Bricks manufacturing	^3^
2009	10	0.220 ± NM	NIOSH7602	East-Tehran	Cement manufacturing	Cement manufacturing	^3^
2011	50	0.29 ± 0.039	NIOSH 7601	Tehran	Metro excavating	Excavations	^34^
2011	25	0.164 ± 0.112	NIOSH7601	-	Glass sandblasting	Sandblast	^35^
2011	5	15.5 ± 0.00	NIOSH7501	Mashhad	Iron stone	Mines	^36^
2012	48	0.34 ± 0.11	NIOSH7602	Mazandaran	Foundry	Foundry	^37^
2012	48	0.19 ± 0.13	NIOSH7602	Mazandaran	Brick industry	Brick industry	^37^
2012	48	0.28 ± 0.10	NIOSH7602	Mazandaran	Sand and gravel production	Sand and gravel production	^37^
2012	48	0.24 ± 0.17	NIOSH7602	Mazandaran	Asphalting	Asphalt industry	^37^
2013	4	0.04 ± 0.02	NIOSH7601	Pakdasht	Foundry	Foundry	^38^
2014	8	0.21 ± 0.19	NIOSH7500	-	Tile industry	Tile and ceramic industry	^39^
2014	60	0.19 ± 0.138	NIOSH7601	Tehran	Demolition of buildings	Construction	^40^
2015	22	0.088 ± 0.055	NIOSH7601	Pakdasht	Foundry	Foundry	^41^
2015	12	0.589 ± 3.04	NIOSH7500	Khuzestan	Cement Company	Cement production	^42^
2016	55	0.246 ± 0.047	NIOSH7602	-	Mining	Mines	^43^
2016	60	0.25 ± 0.13	NIOSH7601	Save	Insulator	Insulator industry	^20^
2016	5	0.13 ± 0.019	NIOSH7602	-	Construction	Construction	^44^
2016	44	0.17 ± 0.79	NIOSH7602	Dorud	Sand washing	Sand and gravel production	^45^
2016	96	0.313 ± 0.180	NIOSH7602	Mazandaran	Sandblasting	Sandblasting	^21^
2016	96	0.169 ± 0.065	NIOSH7602	Mazandaran	Ceramic manufacturing	Tile and ceramic industry	^21^
2016	96	0.282 ± 0.095	NIOSH7602	Mazandaran	Sanding and graveling	Sand and gravel production	^21^
2016	96	0.194 ± 0.130	NIOSH7602	Mazandaran	Brick producing	Brick production	^21^
2016	96	0.239 ± 0.171	NIOSH7602	Mazandaran	Asphalt manufacturing	Asphalt manufacturing	^21^
2016	96	0.338 ± 0.110	NIOSH7602	Mazandaran	Foundry	Foundry	^21^
2016	96	0.125 ± 0.093	NIOSH7602	Mazandaran	Glass manufacturing	Glass manufacturing	^21^
2016	96	0.318 ± 0.120	NIOSH7602	Mazandaran	Stone cutting & milling	Stone deformation operations	^21^
2016	55	0.27 ± 0.11	NIOSH7601	-	Insulator manufacturing	Insulator industry	^46^
2016	114	1.02 ± 0.17	NIOSH7601	East-Iran	Mines	Mines	^47^
2017	40	0.297 ± 272	NIOSH7602	Tehran	Machine brick producing	Bricks manufacturing	^24^
2017	5	0.045 ± 0.03	NIOSH7601	Save	Insulator	Insulator industry	^20^
2017	11	0.052 ± 0.025	NIOSH7601	Save	Insulator	Insulator industry	^20^
2017	14	0.041 ± 0.014	NIOSH7601	Save	Insulator	Insulator industry	^20^
2017	7	0.024 ± 0.008	NIOSH7601	Save	Insulator	Insulator industry	^20^
2017	12	0.039 ± 0.02	NIOSH7601	Save	Insulator	Insulator industry	^20^
2017	127	0.507 ± 0.23	NIOSH7601	Save	Insulator	Insulator industry	^48^
2017	30	0.507 ± 0.23	NIOSH7601	-	Insulator	Insulator industry	^48^
2017	55	0.25 ± 0.05	NIOSH7602	-	Foundry	Foundry	^49^
2017	6	0.17 ± 0.02	NIOSH7602	-	Automobile manufacturing	Machine production	^19^
2018	36	0.12 ± 0.3	NIOSH7500	Khorasan	Cement manufacturing	Cement industry	^50^
2018	55	0.27 ± 0.05	NIOSH7601	-	Insulator	Insulator factories	^51^
2018	5	0.223 ± 0.051	NIOSH7601	-	Furnaces	Furnace	^52^
2018	5	0.218 ± 0.00	NIOSH7601	-	Furnace	Furnace	^52^
2019	40	0.034 ± 0.037	NIOSH7601	Kermanshah	Cement manufacturing	Cement industry	^53^
2020	72	0.027 ± 0.008	NIOSH7602	Nishabur	Concreting	Concrete Workers	^54^
2020	30	0.651 ± 0.69	NIOSH7602	Tehran	Traditional brick producing	Bricks manufacturing	^24^

###  Risk assessment 

 Prior to conducting risk assessment, we investigated the homogeneity of data; moreover, in order to detect and remove outliers, we used the box plot at a 95% confidence level. Mean and geometric standard deviation were calculated for every industrial activity. Thereafter, cumulative exposure to crystalline silica (mg/m^3^-y) (Mean of concentration × Years of exposure) was calculated for risk assessment. The relative risk of death from silicosis was determined using Mannetje’s method.^23^ In this method, the exposure history and crystalline silica concentration are two main factors. In addition, the exposures of all industrial workers in different studies were classified according to the Mannetje category for cumulative exposure.^23^Table 2 displays the exposure-related mortality rates and mortality rate ratios from silicosis in Mannetje’s method.

**Table 2:** Relative risk of silicosis- related mortality in exposed workers according to their cumulative exposure (mg/m^3^-year) to crystalline silica in Mannetje’s method

**Cumulative exposure to crystalline silica (mg/m** ^3^ **-y)**	**Relative risk (95% CI)**
0-0.99	1.00
0.99- 1.97	3.39 (1.42, 8.08)
1.97- 2.87	6.22 (2.56, 15.12)
2.87- 4.33	9.40 (3.71, 23.80)
4.33-7.12	13.69 (5.04, 37.18)
7.12- 9.58	22.64 (7.88, 65.10)
9.58- 13.21	23.97 (8.05, 71.32)
13.21- 15.89	40.25 (13.25, 122.3)
15.89-28.10	25.11 (8.09, 77.91)
> 28.10	63.63 (19.87, 203.8)

 The lung cancer risk of crystalline silica was calculated according to the model of Rice et al^12^ using formula 1. This model is based on the geometric mean of exposure to crystalline silica and 45 years of exposure. In this formula, (A) is the risk of death from lung cancer in workers, and (GM) denotes the geometric mean of exposure to crystalline silica.

 A = 0.77 + 373.69 × GM (1)

## Results

**Figure 1:**
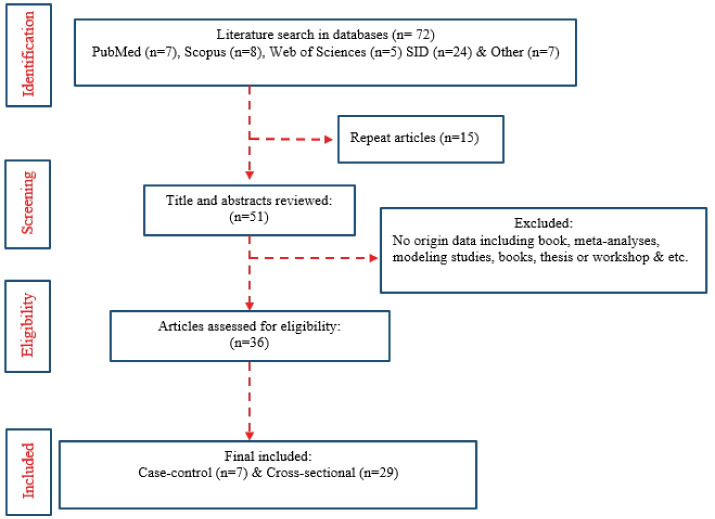
Preferred Reporting Items for Overviews of Reviews displaying selected literature reviews.

 Based on the research reports of the databases, a total of 72 articles were published from September 2000 to September 2020 [PubMed (n = 28), Scopus (n = 8), WOS (n = 5), SID (n = 24), and other databases (n = 7)]. Due to duplication, 15 articles were ruled out. Finally, 36 papers were selected for the study and analyzed by the Preferred Reporting Items for Overviews of Reviews (PRIOR) method (Figure 1). A total of 24 articles were published in Persian. A number of 1, 421 measuring stations in various industries were investigated in 36 studies conducted in the field of worker exposure in Iran. As presented in Table 1, the studies were conducted in eight provinces, including Markazi, Razavi Khorasan, Hamadan, Golestan, Tehran, Mazandaran, Khuzestan, and Lorestan, as well as 13 cities, namely Arak, Save, Kashmar, Khaf, Nishabur, Hamadan, a city in Golestan, Tehran, Pakdasht, a city in Mazandaran, a city in Khorasan, Khuzestan, Dorud. The majority of studies were managed in Razavi Khorasan province (n = 4).

 The studies were performed in 17 industrial activities, such as cement manufacturing, mining, construction, foundry, furnace, stone deformation, glass manufacturing, asphalt manufacturing, sand and gravel production, sandblast, tile, and ceramic industry, brick production, insulator industry, excavation, concrete, food industry, and machine industry. Most studies were performed in mines and foundries (n = 6). One study was carried out in the food industry and another in machine industry. The box plot demonstrated that four studies encompassed outliers. As depicted in Table 1, these data were highlighted by Italic font.^28,33,36,47^ Outliers were deleted and not used in risk assessment; therefore, 2, 036 measuring stations in various industries were selected from 32 studies for risk assessment. In most studies, laborers worked six days a week and their working hours were more than 8 hours.

 The geometric mean concentration for workers’ exposure to crystalline silica ranged from 0.0212-0.2689 mg/m^3^ (Table 3). In addition, the mean concentration of crystalline silica was obtained at 0.1476 ± 0.1628 in Iranian industries, which is higher than recommended exposure standard limit by the American Conference of Governmental Industrial Hygienists (ACGIH), Iran’s national occupational exposure limits (0.025 mg/m^3^),^55^ NIOSH (0.05 mg/m^3^),^56^ as well as OSHA (0.01 mg/m^3^).^57^

**Table 3:** Mean concentrations of exposure to crystalline silica in various industrial activities in Iran

**Industrial activity**	**Number of samples**	**Mean and arithmetic** **standard deviation**	**Mean and geometric** **standard deviation**
Cement manufacturing	98	0.2408 ± 0.2443	0.1516 ± 0.2116
The mines	(77 + NM)^a^	0.2643 ± 0.2171	0.1901 ± 0.1772
Construction	75	0.1710 ± 0.0356	0.1683 ± 0.0290
Foundry	235	0.2332 ± 0.1365	0.1806 ± 0.1246
Furnace	10	0.2205 ± 0.0035	0.2205 ± 0.0025
Stone deformation operations	170	0.3658 ± 0.3565	0.1656 ± 0.3087
Glass manufacturing	106	0.1285 ± 0.0049	0.1285 ± 0.0035
Asphalt manufacturing	154	0.2487 ± 0.0159	0.2483 ± 0.0129
Sand and gravel production	198	0.2483 ± 0.0530	0.2433 ± 0.0459
Sandblast	131	0.2496 ± 0.0769	0.2408 ± 0.0628
Tile and ceramic industry	114	0.2266 ± 0.0674	0.2357 ± 0.0825
Bricks production	189	0.2512 ± 0.2159	0.1605 ± 0.1970
Insulator industry	276	0.2005 ± 0.1912	0.1155 ± 0.1814
Excavations	50	0.2948 ± 0.1169	0.2689 ± 0.1046
Concrete Workers	72	0.0335 ± 0.0311	0.0212 ± 0.0291
Others	81	0.0890 ± 0.1146	0.0368 ± 0.0810
Total	1421		0.1476 ± 0.1628

^a^The number of measuring stations was not mentioned in one of the studies that measured the concentration of silica in the mines.

 As presented in Tables 1 and 2, workers in various industries were exposed to different grades of crystalline silica. In this study, we observed that iron-stone miners were exposed to the highest amount of crystalline silica (Table 1)^33^; nonetheless, to maintain homogeneity, the data related to the study by Naghizadeh et al on iron-stone miners were removed from the risk assessment process. It seems that health risk for iron-stone miners can be at the highest level; therefore, this issue needs more assiduous attention in the future. Furthermore, according to Table 3, workers in excavations are exposed to the highest concentration of crystalline silica (0.2689 ± 0.1046 mg/m^3^). The geometric mean concentration of crystalline silica was the lowest in the food industry (0.008 ± 0.004 mg/m^3^).

## Discussion

 Scarselli et al studied some workers potentially at risk of silica exposure selected from the Italian database of workplaces. They reported that the most involved sectors at high risk of silica exposure were construction, mining and quarrying, metalworking, and manufacturing of non-metallic products. In addition, they reported that workers in the manufacturing and construction industries were exposed to the highest level of crystalline silica.^58^ On the contrary, among the 16 industrial activities classified in this study, the manufacturing and construction industries were the eighth industries.

 In 2015, according to OSHA compliance data from 1979 to 2015, Doney et al reported that workers in the poured concrete foundation had the highest exposure to crystalline silica. Moreover, out of 100 000 workers, 99.7% of cases were potentially exposed to crystalline silica at higher than the occupational exposure limit recommended by NIOSH in 2014.^59^ Concrete workers in Iran had the lowest mean airborne silica exposure levels (0.0212 ± 0.0291), which is lower than the occupational exposure limit recommended by NIOSH, ACGIH, and occupational exposure limits.

 The relative risk of silicosis-related mortality based on Mannetje’s method and cumulative exposure categories was estimated to be in the range of 1-24 per 1000 people, ranging in the cumulative exposure categories of 0-0.99 to 9.58-13.21 in Mannetje’s method. In general, the mean rate of silicosis mortality in Iranian industries was 14 per 1000 people. These rates are above the risk of 1 per 1000 usually deemed acceptable by the US OSHA.^3^

 According to Table 4, the risk of lung cancer due to exposure to crystalline silica based on the Rice model was in the range of 13-137 per 1000 people. In a 44-year cohort study on 34 018 workers, Liu et al reported the risk of lung cancer mortality as 128 per 1000 when the mean cumulative concentration (using a 25-year lag) was 0.01 to 1.12 mg/m^3^-y.^60^ In present study, most investigations were exposed-nonexposed studies. Since risk assessment is calculated by considering the history of exposure to silica, retrospective cohort studies may demonstrate more accurate estimations than other studies. The results of present study demonstrated that the risk of lung cancer was at the highest level among the stone deformation operations (1-137 per 1000). Inconsistent with this finding, Poinen-Rughooputh et al reported that miners were exposed to the highest risk of lung cancer mortality (in the range of 1-104 per 1000).^61^ Moreover, in present study, the lowest risk of lung cancer mortality was estimated in concrete workers in the range of 1-13 per 1000 people.

**Table 4:** Risk of lung cancer mortality in exposed workers in different industries were calculated according to Rice et al. model

**Industrial activity**	**Mean±(SD) (mg/m** ^3^ **)**	**Estimated excess lifetime risks of mortality from lung cancer **
Cement manufacturing	0.2408 ± 0.2443	91
The mines	0.2643 ± 0.2171	100
Construction	0.171 ± 0.0356	65
Foundry	0.2332 ± 0.1365	88
Furnace	0.2205 ± 0.0035	83
Stone deformation operations	0.3658 ± 0.3565	137
glass manufacturing	0.1285 ± 0.0049	49
Asphalt manufacturing	0.2487 ± 0.0159	94
sand and gravel production	0.2483 ± 0.0530	94
Sand blast	0.2496 ± 0.0769	94
Tile and ceramic industry	0.2266 ± 0.0674	85
Bricks production	0.2512 ± 0.2159	94
Insulator industry	0.2005 ± 0.1912	76
Excavations	0.2948 ± 0.1169	111
Concrete Workers	0.0335 ± 0.0311	13
Others	0.0368 ± 0.081	15

 In 2016, in a meta-analysis study, based on worldwide studies up to April 2016, the highest pooled concentration mortality ratio of exposure to crystalline silica was estimated at 6.03 (95% CI: 5.29-6.77) in mixed industries of Japan. Moreover, Italy had the highest number of observed lung cancer deaths (798 cases) before 2006.^61^ In their study, although the estimated health risk was high in Asian countries after Canada, the studies in Iran have been neglected. According to the results of the current research, the risk of both silicosis and lung cancer mortality is high in Iranian industries, and even numerous studies were conducted before 2016 in Iran.

 Among the notable limitations of this study, we can refer to incomplete information on workers of all industries; therefore, we could not estimate the risk of mortality based on the percentage of exposed workers in industries. The expression of mortality based on the percentage of exposed cases can provide a better understanding of the hazard. Due to the high risk of silicosis and lung cancer mortality, it seems that the prevalent occupational health engineering strategies are not sufficient to protect workers; therefore, workers’ exposure to crystalline silica dust should be controlled in Iranian workplaces.

## Conclusion

 The authors provided a lung cancer risk assessment of occupational exposure to crystalline silica in Iranian industrials based on the collected quantitative exposure data. As evidenced by the obtained results, occupational exposure to crystalline silica was higher than occupational exposure limits. Furthermore, the relative risk of death from silicosis was in the range of 1-24 per 1000 people, and the risk of lung cancer ranged from 13-137 per 1000 people. It seems that the prevalent occupational health engineering strategies are not sufficient to protect workers; therefore, workers’ exposure to crystalline silica dust should be controlled in Iranian workplaces.

**Highlights**

- The present study estimated silicosis and lung cancer caused by crystalline silica.
- Workers’ exposure to crystalline silica ranged from 0.0212-0.2689 mg/m3.
- This range was higher than the recommended standard limit by ACGIH (0.025 mg/m3).
- The relative risk of silicosis mortality was in the range of 1-14 per 1000.

## Acknowledgments

 We would like to express our special appreciation for Dr. Mohammad Asghari Jafarabadi, Professor of Biostatistics, Tabriz University of Medical Sciences, Iran, who helped to analyze data in this study. Their willingness to give their time so kindly is very much appreciated.

## Conflicts of Interest

 The authors declare that they have no conflict of interest.

## Ethical Approval

 The study was based on the literature search and was not an experimental study; therefore, there was no need for ethics committee approval.

## Funding

 No funding was received.
